# MOLGENIS/connect: a system for semi-automatic integration of heterogeneous phenotype data with applications in biobanks

**DOI:** 10.1093/bioinformatics/btw155

**Published:** 2016-03-21

**Authors:** Chao Pang, David van Enckevort, Mark de Haan, Fleur Kelpin, Jonathan Jetten, Dennis Hendriksen, Tommy de Boer, Bart Charbon, Erwin Winder, K. Joeri van der Velde, Dany Doiron, Isabel Fortier, Hans Hillege, Morris A. Swertz

**Affiliations:** ^1^Department of Genetics, University Medical Center Groningen, Genomics Coordination Center, University of Groningen, Groningen, The Netherlands; ^2^Department of Epidemiology, University Medical Center Groningen, University of Groningen, Groningen, The Netherlands; ^3^Research Institute of the McGill University Health Centre and; ^4^Department of Medicine, McGill University, Montreal, Canada

## Abstract

**Motivation:** While the size and number of biobanks, patient registries and other data collections are increasing, biomedical researchers still often need to pool data for statistical power, a task that requires time-intensive retrospective integration.

**Results:** To address this challenge, we developed MOLGENIS/connect, a semi-automatic system to find, match and pool data from different sources. The system shortlists relevant source attributes from thousands of candidates using ontology-based query expansion to overcome variations in terminology. Then it generates algorithms that transform source attributes to a common target DataSchema. These include unit conversion, categorical value matching and complex conversion patterns (e.g. calculation of BMI). In comparison to human-experts, MOLGENIS/connect was able to auto-generate 27% of the algorithms perfectly, with an additional 46% needing only minor editing, representing a reduction in the human effort and expertise needed to pool data.

**Availability and Implementation:** Source code, binaries and documentation are available as open-source under LGPLv3 from http://github.com/molgenis/molgenis and www.molgenis.org/connect.

**Contact**: m.a.swertz@rug.nl

**Supplementary information:** Supplementary data are available at *Bioinformatics* online.

## 1 Introduction

Biobanks, patient registries and other human data collections have become an indispensable resource to better understand the epidemiology and biological mechanisms of disease. While these collections have grown to include data from over 100 000s of individuals (Scholtens *et al.*, 2015), many research questions still require data from multiple collections to reach sufficient statistical power or to achieve sufficient numbers of subjects having rare (disease) characteristics. To make data integration easy, all collections would ideally use the same data collection protocols and questionnaires. In practice however, biobanks collect different data because of differences in their scientific goals. For integration to be valid, data must be compared and harmonized before combined analyses are carried out (Fortier *et al.*, 2011).

Substantial efforts are now underway to make data ‘inferentially equivalent’ or ‘harmonized’ as a basis for pooled analysis. The Maelstrom Research group has taken the lead in defining protocols for retrospective data integration (https://www.maelstrom-research.org/) (Maelstrom Research, 2015). Within the BioSHaRE project, we have re-used and refined this protocol to harmonize and integrate 90 variables from 9 biobanks as a basis for pooled analysis (Doiron *et al.*, 2013). This research-question-driven approach consists of three steps:

1. *Defining the target DataSchema*: the list of targeted variables necessary to address the research questions in a specific study;
2. *Matching biobank schemas to the target DataSchema*: match data elements from participating data sources/biobanks to the variables in the target DataSchema;
3. *Generating of Extract-Transform-Load algorithms*: define the algorithms that take the matched source data elements as the input and convert these data values to the target DataSchema for data integration.

Existing biomedical data integration tools still require significant manual effort and technical skill. For example, Maelstrom uses Opal software for biobank pooling with a professional team to find mappings and create algorithms, available at http://www.obiba.org/pages/products/opal/ (Opal, 2011). Similarly, clinical/translational data warehouses tranSMART and i2b2 require knowledgeable analysts to manually identify mappings, based on which ETL developers implement the programmatic transformations (Murphy *et al.*, 2010; Szalma *et al.*, 2010).

To alleviate this burden, we previously presented BiobankConnect, a system to semi-automatically match data elements from biobanks to target variables (Pang *et al.*, 2015a). In this paper, we introduce an additional system to semi-automatically define the transformation algorithms to produce an integrated dataset. We have wrapped all functions described above into an integrated user interface, MOLGENIS/connect, to support research teams through the entire integration procedure.

## 2 Methods

**Fig. 1.**
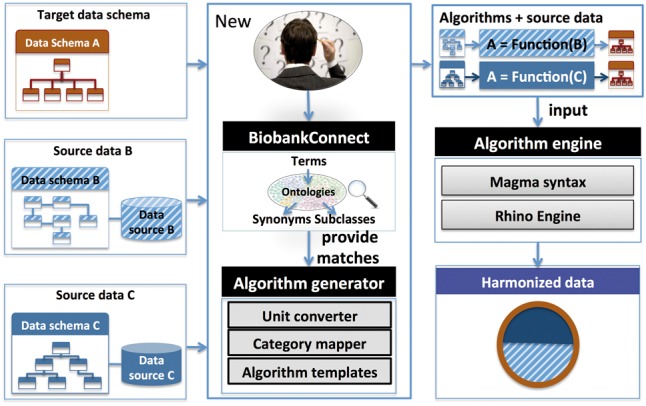
The overview of the framework of MOLGENIS/connect

We have used the Maelstrom Research harmonization protocol as the basis for our system. Figure 1 provides an overview of its main components. First, we implemented a metadata model component that allows users to upload, view and visualize the target DataSchemas as well as the data of the source biobanks. Second, we incorporated a semantic search facility to shortlist candidate source data element matches to each variable in the target DataSchema. Third, an integration algorithm generator incorporates algorithm templates, semantic searches, category convertors and a unit convertor.

### 2.1 Metadata model

**Fig. 2.**
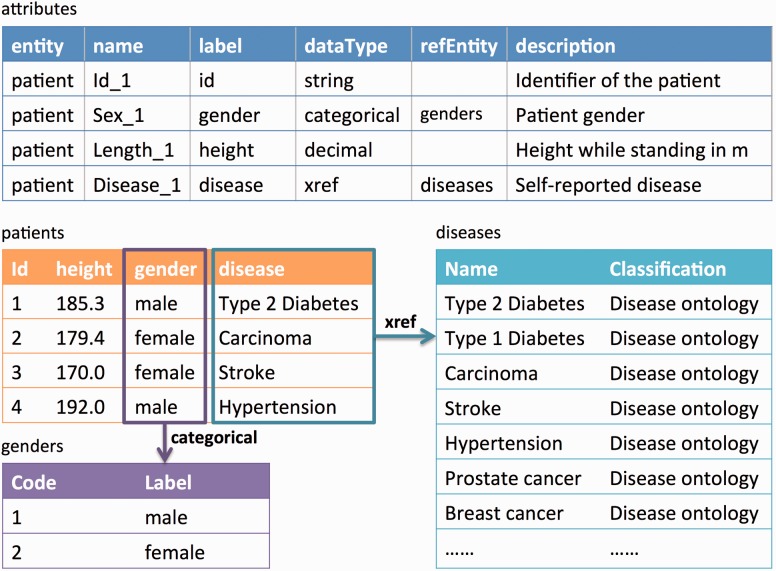
Example of the EMX data upload format. Data can be uploaded using Excel Metadata describing the columns of each data sheet (i.e. ‘entity’) that must be provided in a special ‘attributes’ sheet. Data values are stored in ordinary sheets (e.g. ‘patients’). The ‘categorical’ gender attribute and the ‘xref’ disease attribute refer to another two sheets, ‘genders’ and ‘diseases’ (omitted for readability)

To load both the target DataSchema as well as the various biobank data models (i.e. data dictionaries), we have designed a flexible meta-model called Entity Model Extensible (EMX), the documentation is available at http://molgenis.github.io/documentation/ (Molgenis, 2014). [TQ1]This model evolved from Observ-OM, which has been proven to model all kinds of biomedical data (Adamusiak *et al.*, 2012). EMX is a lightweight version of Observ-OM in which only two types of information (Entity and Attribute) are needed to sufficiently describe a dataset. Attributes are features that can be observed such as ‘disease’, ‘gender’ and ‘height’, and which are often referred to as ‘metadata’ by researchers. In EMX, an attribute ideally contains the following information: a unique name, a pre-defined data type (e.g. string, integer, decimal), a human readable label, a detailed description of the attribute and how it can be used, and categories or cross-references (xrefs) if the data type is categorical or a relationship (e.g. ‘Gender attribute’ has two categories, ‘Male’ and ‘Female’). Entities are definitions of tables that define groups of attributes as columns and data (entity instances) as rows. The relations of entities and attributes are described in Figure 2. In the rest of this paper, we will refer to both of the variables of the target DataSchema and the data elements of the source (biobank) as ‘attributes’.

### 2.2 Semi-automatic source-to-target attribute matching

Standard practice for identifying candidate biobank attributes for pooled analyses has been to manually go through all data attributes of all biobanks, an extremely time-consuming process. To automate this step, we used our previously published BiobankConnect method (Pang *et al.*, 2015a). It combines the Information Retrieval System of Lucene, available at https://lucene.apache.org/core/ (The Apache Software Foundation, 2006), with query expansion to automatically shortlist good candidate attributes. It consists of (i) query expansion (Bhogal *et al.*, 2007) in which attributes of the target DataSchema are (semi-) automatically annotated (‘expanded’) with ontology terms, whose synonyms and subclasses are collected to create a list of semantically identical or similar terms that get added to the original query to find other relevant source attributes and (ii) retrieving relevant attributes in which the ‘expanded’ target attributes are matched against the biobank attributes using Lucene, and matched candidates are sorted based on Lucene scores for human experts to choose from, as described in (Pang *et al.*, 2015a).

### 2.3 Transformation syntax

To create an executable data integration procedure, the rules for transforming data from source to target attributes need to be encoded in a computer algorithm. These algorithms transform attribute values from the source datasets to the statistically equivalent attribute value required in the target DataSchema. The simplest algorithm simply renames the source attribute, e.g. transforming ‘length’ (in LifeLines) to ‘height’ in the target DataSchema. More advanced algorithms can implement unit conversions, recode categories or execute more advanced formulas like a body mass index (BMI) calculation.

For the implementation of the transformation algorithms, we have used the ‘Magma’ (Magma, 2014) syntax, available at http://wiki.obiba.org/display/OPALDOC/Magma+Javascript+API, which is a domain-specific programming language for data harmonization that was used in BioSHaRE. Magma is a JavaScript library that works similar to jQuery, a popular JavaScript framework. To access values, the name of attributes can be wrapped in brackets and a dollar sign, e.g. $(‘var’). There are many methods available in Magma which can be called by chaining calls to the attribute accessor, e.g. $(‘var’).div(2). We have implemented the most commonly used methods including div(), times(), plus(), map(), pow(), unit() and toUnit(). In addition, we have created an algorithm generator, which consists of a unit conversion algorithm generator, a categorical values algorithm generator and a complete algorithm generator, described below.

### 2.4 Unit conversion algorithm generator

One of the recurring challenges in data harmonization is harmonizing units. Detecting units in attribute metadata can be difficult because different forms of units are used to describe the same parameter in different databases, e.g. ‘meter’ is used to describe the attribute ‘Height in meter’ in one database while ‘cm’ is used in describing the attribute ‘Body length in cm’ in another. Because no suitable algorithm generator could be found, we have developed a new two-step method for unit convertor generation. First, unit terms that occur in the label of target attributes and/or source attributes are annotated with the Units of Measurement Ontology (UO). Labels of attributes and target attributes are tokenized by whitespace and matched against terms in the UO using Lucene (analogous to how BiobankConnect does attribute matching). To prevent false positives, we accept only exact matches for unit detection. Second, we have used the unit converter software library developed by JScience (JScience, 2012), which is implemented based on The Unified Code for Units of Measure http://www.unitsofmeasure.org/trac (Schadow and McDonald, 2005), for international standard units and commonly used non-standard units, available at http://jscience.org/. This has a list of conversion rules for units that are compatible, e.g. cm = m × 100 or g = kg × 1000. For example, to convert units from ‘centimeter’ to ‘meter’ for the attribute ‘Height’, the terms ‘centimeter’ and ‘meter’ are automatically annotated with ontology terms UO:centimeter and UO:meter, respectively, based on the formal name and synonyms of the units. The formal symbols of these two units (cm and m) collected from the UO are then parsed to JScience, in which the suitable rule is found for converting ‘cm’ to ‘m’ and incorporated into the algorithm template. We implemented two different syntaxes for unit conversions: using a chain of explicit methods, e.g. **$(‘Height’).unit(‘cm’).toUnit(‘m’).value()**, or more by generating the necessary calculation formula, e.g. **$(‘Height’).div(100).value()**. In the case of composite units or derived units such as kg/m^2^, we first break them into the smallest units (atomic units), then compare the atomic units with units of matched attributes individually, and finally convert the units accordingly. For example, the target attribute BMI (kg/m^2^) is matched to source attributes **height in cm** and **weight in gram**. The term kg/m^2^ is broken apart into a set of atomic units, kg and m, which become the standard units because they are detected/derived from the target attribute, the cm and gram units detected from source attributes are then converted accordingly.

### 2.5 Categorical values matching generator

Another recurring challenge is to generate algorithms that convert between categorical values. For this, we explored matching categories automatically and identified three different types of categories that need to be matched:

- *Matching categories using lexical similarity*: To find lexically similar categories, we calculate the pairwise n-gram similarity scores between all target and source categories. For each source category, the target category that yielded the best n-gram similarity score is automatically selected as the best match. For example, the target attribute (Gender: ‘0 = Male, 1 = Female’) and the source attribute (SEX:‘1 = Male, 2 = Female’) have the same category labels but different category codes, the system matches two sets of category labels onto each other based on the n-gram-based string matching algorithm and with the final result $(‘Gender’) = $(‘SEX’).map({‘1’: ‘0’, ‘2’: ‘1’}). Thus source category 1 and 2 are matched to target category 0 and 1, respectively.
- *Matching categories that represent frequencies:* After scrutinizing many biobank attributes and the target attributes, we realized that there are a class of attributes that describes the frequencies of certain activities or food consumption. Supplementary Table S1 shows an example of matching attributes for consumption of potatoes. The categories contain two types of information, time units and frequencies, which can be extracted using regular expressions, e.g. 2–4 times a week has an average frequency 3 (2–4) and the time unit week. The first step is to convert both the target and source categories to quantifiable amounts; the second step is to find the closest target amount category for each source amount category. Because categories are often not matched one-to-one, the algorithm is allowed to have multiple source amounts matched to one target amount. The matching category function is implemented in Java using JScience library (JScience, 2012).
- *Matching categories based on pre-defined rules:* In Supplementary Table S2, we show a list of custom rules for matching categories that we have hard-coded into the system.

### 2.6 Overall algorithm generator

The creation of algorithms is a tricky task and nearly impossible for those inexperienced in programming. Therefore, as a last step, we created a generator that assembles the complete algorithms. Moreover, we have provided a catalogue of templates for more complex algorithms such as ‘BMI calculation’, which can be found in the Supplementary material **javascript_magma.xls**. Each template defines its source and target attributes. These matching templates will be proposed to the user if one or more of the matched attributes relates to this template, e.g. ‘height’ or ‘weight’ in the case of BMI.

**Fig. 3.**
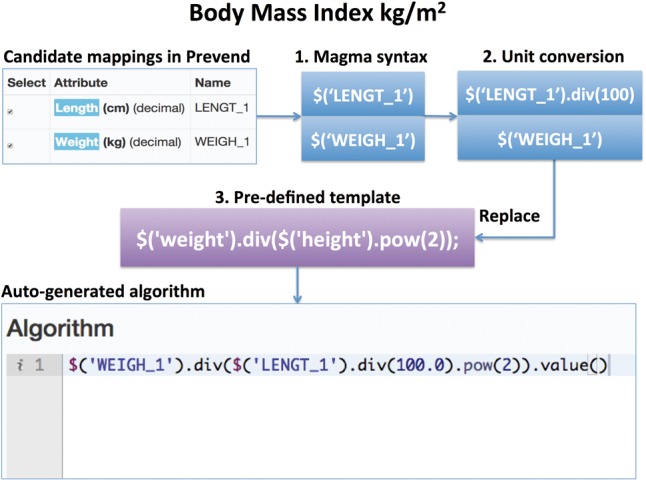
Example of algorithm generation for target attribute BMI from the Prevend data source (1) a transformation template is generated from the candidate matches (using Magma syntax), (2) the template is automatically edited based on unit conversion rules if applicable and (3) the software evaluates if more complex algorithm templates can be used. Based on two good candidate matches and the desired ‘BMI’ target, a previously used BMI conversion algorithm is proposed that incorporates the unit conversion rules (e.g. from ‘cm’ to ‘m’ because BMI is recorded as composite unit kg/m^2^)

Figure 3 summarizes the process of generating the complete algorithm using the example of the target attribute ‘Body Mass Index’ from source biobank Prevend is summarized. It consists of the following steps: I) the system looks in its database to find the available algorithm template for BMI, II) it uses the BiobankConnect algorithm to generate a list of relevant attributes, III) it applies the unit conversion algorithms towards kg/m^2^ (e.g. LENGT_1 was measured using centimeter (cm) rather than the standard unit meter (m) and therefore needs to be converted), and IV) the building blocks within the BMI template are replaced with the matched attributes using the string-matching algorithm (n-gram)(e.g. ‘weight’ was matched with ‘WEIGHT_1:Weight (kg)’ and ‘height’ was matched with LENGT_1: Length (cm) based on the best lexical similarity scores).

## 3 Implementation

We have implemented above methods into a seamless user workflow: (i) users upload a target DataSchema and the source biobank data, (ii) users then create a mapping project and select target DataSchema and data sources, (iii) MOLGENIS/Connect automatically generates all matches and conversion algorithms for all data sources and all target attributes, (iv) the user curates each of the matches and algorithms using the algorithm editor and preview tool and (v) MOLGENIS/Connect generates the integrated dataset. We describe each step in detail below. The integration tool has been built on top of the MOLGENIS software suite and reuses its basic functions (upload, metadata viewer, data explorer, permission system) (Swertz *et al.*, 2010). MOLGENIS is a Java/Maven web application implemented using MySql and ElasticSearch as back-end and HTML5, Bootstrap, jQuery, ReactJS as front-end. The source code is available at https://github.com/molgenis.

### 3.1 Upload and view target DataSchema and data sources

In this step, users upload target DataSchema and source data via the standard MOLGENIS upload. For this purpose, we use the ‘EMX’ format (Molgenis, 2014), a spreadsheet-based format to describe and upload tabular datasets and definition of their schemas that can be edited directly using Microsoft Excel or text editor (CSV files). For the target DataSchema, one spreadsheet is required that defines ‘attributes’ of the target DataSchema such as name, description and data type (see ‘attributes’ sheet in Fig. 2). For each biobank, two spreadsheets are required: a ‘attributes’ metadata sheet just like the target DataSchema that defines the attributes of each dataset and one or more dataset sheets where each column matches the attributes and each row is, e.g. data on each biobank participant (see ‘your data’ table in Fig. 2). The data that has been uploaded can be viewed and filtered using MOLGENIS data explorer.

### 3.2 Create a mapping project

**Fig. 4.**
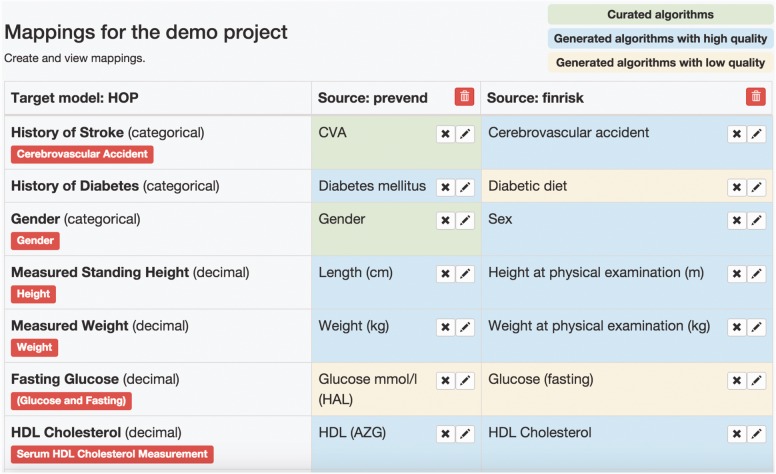
Mapping project overview. The attributes of the target DataSchema are shown on the left of the table. The columns contain matching attributes from each of the sources. New source data can be added by clicking the ‘+Add source’ button. Attribute matches and conversion algorithms are automatically generated and colour coded to indicate if the algorithms are generated with high confidence (perfect match in semantic search) or low quality (partial match in semantic search) or to indicate if an algorithm has been curated by the user

In this step users start a new mapping project with the desired DataSchema as the target and the biobank datasets as the sources. Once these are selected, the system will generate an overview of attribute matches (described below) (Fig. 4).

### 3.3 Generate overview of attribute mappings from source to target DataSchema

In this step the system generates a complete overview of all target attributes (shown in the first column) and all the matches from the source attributes (shown in the following columns), see Figure 4.

When a user selects a new data source, the system automatically generates candidate matches. Each match can be edited and tested using the algorithm editor described below. To open this view, users click on the pencil icon located in any of the cells. For this purpose, we have refactored the BiobankConnect system, which uses ontology terms to generate the candidate matches (Pang *et al.*, 2015a). Based on user feedback, we learned that manual annotation of target attributes with ontologies previously required was too labour-intensive. We have, therefore, now included automatic annotation in which the label and description of the target attributes are used to find ontology terms in all available ontologies (e.g. NCI, SNOMED CT and MeSH) in the database.

### 3.4 Edit and test data transformations

In this step the user can edit the integration algorithm, see Supplementary Figure S3. This is the heart of the system and consists of three components: (i) the source attribute selector, (ii) the algorithm editor and (iii) the result preview.

In the *source attribute selector* (shown on the left of the screen) shortlists candidate attributes sorted by lexical matching scores between the ontology terms associated to the target attribute and label or description of the source attributes. The words from the ontology terms are highlighted in each attribute label or description. Based on the importance of the highlighted words, users can immediately determine whether the candidates generated are good matches for the target attribute or not. In the example in Supplementary Figure S4a, the words *blood* and *pressure* are highlighted in the attribute ‘Mean blood pressure’ and it is clear that this attribute is related but not the same as ‘Hypertension’. If no good candidates are shown, the user can enter terms in the semantic search box to quickly find additional attributes using the syntax **term1 or term2** (e.g. weight or gender), see Supplementary Figure S4b. These query terms are matched with ontology terms to enable expanded query.

In the *algorithm editor* (shown in the middle), the user sees the auto-generated algorithm for the selected attribute (or multiple attributes) using the Magma/JavaScript syntax (see methods section). We mostly dealt with two types of target attributes: numeric attributes whose value can either be integer or decimal, e.g. the value for ‘height’ is a decimal number, and categorical attributes which only have a limited number of allowed values, e.g. values for ‘gender’ written in the JSON-like (http://www.json.org/) (JSON.org, 2014) format {code = 0, label = male}, {code = 1, label = female}. To generate algorithms for these target attributes, we usually need one source attribute, although sometimes the values of multiple attributes need to be combined, e.g. values for ‘BMI’ must be generated via ‘height’ and ‘weight’. Other data types supported include Date, Boolean, String and Text (see EMX documentation).

In the *result preview* (shown on the right of the screen), the user sees a subset of the results of the converted data and how many of the data conversions failed, e.g. because of syntax errors. This allows users to rapidly test and correct their conversion algorithms.

### 3.5 Create the derived dataset and explore the results

Having defined the algorithms in Magma/JavaScript as described above, users can execute the transformation process from within the mapping project overview. The data conversion engine is implemented using Rhino and the R interface with Rcurl and rjson, where Rcurl is used to retrieve data in JSON (JSON.org, 2014) format and convert it to a DataFrame object in R. A new dataset is then created that stores values in the target DataSchema. Users can access the data through MOLGENIS data explorer where advanced filtering function and visualization capability are offered. The integrated data can be downloaded in comma-separated values (CSV) and Microsoft Excel. We also provide the R Application Programming Interface (R-API), which allows users to access data in the R statistical environment (see MOLGENIS documentation), and HTTP REST/JSON interfaces to integrate with other software.

## 4 Results

We performed a qualitative evaluation by applying the software in active BioSHaRE, BBMRI and RD-Connect harmonization projects and a quantitative evaluation by comparing the auto-generated algorithms with the manually curated algorithms within the BioSHaRE Healthy Obese Project (Van Vliet-Ostaptchouk *et al.*, 2014).

### 4.1 Matching numeric attributes

In the example shown in Supplementary Figure S5a, the target attribute ‘Measured Standing Height’ was matched to source attributes in the LifeLines biobank (Scholtens *et al.*, 2015). The first source attribute suggested, ‘Height in cm’, is used by default in generating the algorithm. The unit ‘cm’ was detected by the system in the source attribute whereas there was no mention of unit in the target attribute, therefore the target unit was assumed to be the same as the source attribute and unit conversion was not needed. Algorithms are executed automatically whenever users change the algorithm syntax in the editor; an updated preview of algorithm results is provided to evaluate.

### 4.2 Matching categorical attributes

Supplementary Figure S5b shows another example, in which the target attribute and the source attribute were both categorical. We implemented the Magma map({c1:c1’, c2:c2’….}) function to match categories of the target attribute and source attribute onto each other. A category-matching editor is demonstrated, where two sets of categories could be easily matched by selecting target categories from the dropdown menus. The results from the matching editor were converted to the Magma syntax so users could easily create matching functions without writing complex algorithms.

### 4.3 Evaluation of algorithm generator

We compared the output of the auto-generated transformation algorithms with manually curated algorithms for all 90 target attributes from the BioSHaRE Healthy Obese Project (Van Vliet-Ostaptchouk *et al.*, 2014) and three of the biobanks (LifeLines, Prevend and Mitchelstown) for which we had the participant-level data values (184 algorithms in total). We evaluated the performance of semantic search and algorithm generation separately.

To evaluate the semantic search, we defined three result categories: perfect search, good search and bad search. A search result is ‘perfect’ when the human-matched source attribute was ranked 1^st^ in the system-suggested list. A search result is ‘good’ when all human-matched source attributes can be found within top 20 of the suggested list. We chose this threshold because there were a few target attributes for which HOP research assistants used more than 10 source attributes. For example, there are 16 source attributes related to the target attribute ‘current consumption of meat product’ in Mitchelstown.

To evaluate the algorithm generator, we also defined three categories (perfect, good and bad). Algorithms were classified as ‘perfect’ when the auto-generated algorithms were the same as or functionally equivalent to manually created ones (i.e. when the algorithms yield the same target values when executed on the source dataset). Algorithms were ‘good’ when they were almost correct but still required the users to fix them by hand. For example, when half of the categorical values were correctly matched between the source and the target attributes, but some additional matches also needed to be added by hand to complete the algorithm. An algorithm is evaluated to be ‘bad’ when the algorithm needs to be completely replaced by a human-edited version.

Table 1 summarizes the quantitative evaluation (the complete data can be found in the Supplementary material **Evaluation_results.xlsx)**: 27.7% of the algorithms generated were immediately equivalent to the manually created ones (perfect search, perfect algorithm); 9.8% of the algorithms generated where perfect, but only after users chose the right source attributes from the list of candidates (good search, perfect algorithm); 16.8% of the algorithms generated were partially correct and required users to modify them (perfect search, good algorithm); also we considered (good search, good algorithm), (bad search, perfect algorithm) and (perfect search, bad algorithm) to be useful. Thus, in total, 73% of the results were deemed useful (summing up the green colour-coded cells in Table 1, 27.7 + 16.8 + 1.6 + 9.8 + 7.1 + 18 = 73).

**Table 1.** Summary of the quality measures of algorithm generator and semantic search (in percentages)

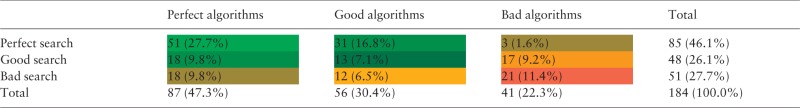

Cells are colour-coded to represent the amount of human input (manual work) required to fix the matching, with green being the easiest and red being the most difficult (Please see the online article at http://bioinformatics.oxfordjournals.org/ for the colour-coded table).

## 5 Discussion and future work

In the RESULTS section we demonstrated that MOLGENIS/connect can help users can quickly identify relevant source attributes and that the program auto-generates mostly useful data integration algorithms. Here we discuss potential areas of improvement.

### 5.1 Domain-specific improvements

**Fig.5.**
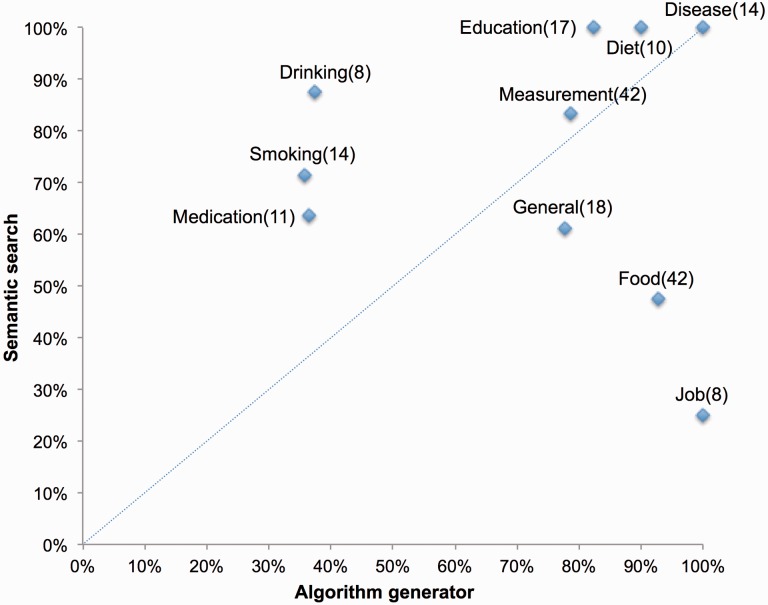
Scatter plot visualizing the success rates of algorithm generator and semantic search per attribute domain. The *X*-axis and *Y*-axis represent ‘useful algorithm’ (defined as when the algorithms generated are correct or partially correct) and ‘useful search’ (defined as when the matched source attributes found fall within top 20 of the suggested list) categories of algorithm generator and semantic search in Table 2. The numbers in parenthesis are the number of attributes for the corresponding topics

To obtain more insights into the cases for which the system performs well and the cases for which the system needs improvement, we have grouped all the target attributes into 10 areas of information: Diet, Disease, Alcohol use, Education, Food, Employment, physical and laboratory measurement, Medication, Tobacco use and General (e.g. Age, Gender). We summarize the performance of the algorithm generator as well as semantic search per topic in Table 2 and Figure 5, for further details see Supplementary Table S6.

**Table 2.** Quality measures of algorithm generator and semantic search in percentages, grouped by attribute topic

	Algorithm generator			Semantic search		
	Perfect (%)	Good (%)	Bad (%)	Perfect (%)	Good (%)	Bad (%)
Diet (10)	50	40	10	70	30	0
Disease (14)	86	14	0	71	29	0
Drink (8)	0	38	63	50	38	13
Education (17)	0	82	18	65	35	0
Food (42)	88	5	7	14	33	52
General (18)	28	50	22	50	11	39
Job (8)	0	100	0	25	0	75
Measurement (42)	62	17	21	74	10	17
Medication (11)	0	36	64	27	36	36
Smoking (14)	1	21	64	14	57	29
Total (184)	47	30	22	46	26	28

The numbers between brackets indicate the number of target attributes.

Figure 5 indicates that semantic search does not perform well on ‘Food and ‘Job’ while algorithm generator needs improvement for ‘Medication’, ‘Smoking’ and ‘Drinking’. Smoking and Drinking turned out to be very difficult to handle because how these attributes are defined in different biobanks varies in description and structure. There are more than 40 smoking-related attributes in LifeLines versus only 3 in Prevend. As a consequence, it was very difficult for semantic search to identify ‘the one attribute’ among many similar ones. Further, because there were few recurring patterns, the algorithm generator did not know how to generate the algorithms even though the source attributes were provided. We originally thought that the attribute Medication would be well standardized across biobanks due to the use of ATC code. In practice, some biobanks still use internally defined terminology to record medication information, making it more challenging to integrate medication data automatically. On the other hand, rather complex Food and Job target attributes scored unexpectedly ‘good’ in algorithm generation.

Semantic search is currently limited because we only used small subsets of SNOMED CT and NCI Thesaurus ontologies (for performance reasons). The search capability may be further improved by using the complete version of those ontologies. For instance, the target attribute ‘Current Consumption Frequency of Poultry and Poultry Products’ was matched to the source attribute Breaded chicken through manual matching, but semantic search missed this match due to the lack of knowledge of such terminology. The relation ‘Chicken is_subclass_of Poultry’ is stated explicitly in full SNOMED CT and search results could be greatly improved by incorporating such information. Other challenges in mapping attributes are the problem of family history, e.g. ‘parental diabetes’ which was discussed in (Pang *et al.*, 2015a), and of negation, e.g. ‘I do not smoke’ is considered relevant to the target attribute ‘quantity of cigarette smoked’. One of the potential solutions would be to highlight the negative words in a specific colour in the suggested source attributes, such as not, never and do not, so users can immediately choose to skip those attributes.

### 5.2 Complex algorithms

Although semantic search and algorithm generator seem to work well, the algorithm template functionality is still limited because we can only define templates for target attributes that have a clear definition or recurring pattern such as BMI and hypertension. It is not possible to formulate templates for ambiguous target attributes. For example, BioSHaRE researchers manually created the algorithm for the target attribute *Quantity of Beer Consumption* in LifeLines following the logic (i) whether or not the participants have had any alcoholic drinks (yes/no); (ii) if ‘yes’ the quantity of beer will be returned otherwise a null value will be returned. The pseudo code of the algorithm is shown below:

$$\begin{array}{l} \text{if(\$(‘drinking\_alcohol’).value()== ‘yes’)}\\ \text{ return \$(‘beer\_quantity’).value();}\\ \text{else}\\ \text{ return null;} \end{array}$$

However, there are two major remaining challenges in generating this kind of algorithm. First, semantic search is only able to find beer-related attributes; it still misses the alcohol-drinking-related ones because, while subclass relations are used in the query expansion in semantic search, reversed relations are not. The search knows about the fact that **beer** is a *subclass_of* **alcoholic drink** but does not understand that **alcoholic drink** is a *superclass_of* **beer**. We did not include such reversed relations in the query expansion to prevent semantic search from finding too many false positives (irrelevant source attributes). This problem could be solved in the future by including a ‘semantic relatedness’ metric into the system. Wu and Palmer proposed to calculate the semantic similarities of any two concepts by considering the depths of the concepts within the ontological hierarchy and the lowest common ancestor in the WordNet taxonomy (Wu and Palmer, 1994),

$$\text{WUP}\_\text{similarity}=\frac{2\times \text{depth}\_\text{of}\_\text{lowest}\_\text{common}\_\text{ancestor}}{\text{depth}\_\text{of}\_{\text{concept}}^{1}+\text{depth}\_\text{of}\_{\text{concept}}^{2}}$$

For example, the semantic similarity for ‘beer’ and ‘alcoholic drink’ is 91% when using the tool provided by wsj4 Java library online demo http://ws4jdemo.appspot.com/?mode=w&s1=&w1=beer%23n%231&s2=&w2=alcoholic_drink%23n%231 (Shima, 2011).

Second, even if suitable source attributes (**beer** and **alcoholic drinks**) can be found by semantic search, the algorithm generator does not know how to handle them because there are no suitable templates for these two attributes. One of potential solutions would be to train the system to learn the patterns of the existing algorithms defined by the human experts, i.e. to reuse all the matches that have been created before as potential templates. This would enable the system to utilize the human expert knowledge now implicitly available in the data conversion algorithms.

### 5.3 Repeated measurements

We observed that the same attribute is often measured multiple times to reach a high precision or to establish time series. For instance, in the Mitchelstown biobank, systolic blood pressure was measured three times: *systolic blood pressure 1^st^ reading*, *systolic blood pressure 2^nd^ reading* and *systolic blood pressure 3^rd^ reading*. When the target attribute *Systolic Blood Pressure* is matched to Mitchelstown, we could decide to take the average value of those source attributes. Because all the repeated attributes are lexically close, it would be possible for the system to check if the top suggested attributes are repeated measurements and then decide whether or not to take the average value.

### 5.4 Matching and recoding of categorical data

To robustly match categories, we not only enabled lexical matching but also developed a new frequency matching method (see Supplementary Table S1). Moreover, we introduced a rule-based category matching system in which we have hardcoded rules to make the system smart enough to deal with difficult categories (see Supplementary Table S2). Most of the categories shown in the evaluation section could be matched correctly, but there will no doubt be new special cases that require us to add new rules. We would like to allow users to define custom rules for matching categories in the database. For matching string-type data values, we have developed a tool (SORTA) to semi-automatically recode the values based on the selected coding systems or ontologies, which we plan to incorporate in the near future (Pang *et al.*, 2015b).

### 5.5 Statistical matching

Although units are now accurately detected from the label of attributes using the string-matching algorithm, not all attributes actually contain any information regarding units. In those cases, users now have to guess the unit from data values based on their empirical experience. However, when biobank datasets are available in the system, it should be possible to extrapolate the units using a statistical approach in which the distribution of data values is compared to the distributions of other source data values for which unit information is available.

## 6 Conclusion

We have introduced and demonstrated the utility of MOLGENIS/connect, a generic computer system for semi-automatic harmonization and integration of data with focus on human phenotypes in biobanks, patient registries and biomedical research. The system includes a novel method to automatically generate harmonization/integration algorithms based on ontological query expansion, lexical matching and algorithm template matching. Evaluation in 184 BioSHaRE matches showed MOLGENIS/connect is able to generate useful matches and algorithms in 73% of the cases while only 11% still needed to be created by completely hand. Users can use these auto-generated algorithms to rapidly design and execute the integration via a user-friendly online web application. The application and source code are available as open source via the MOLGENIS software suite at http://github.com/molgenis/molgenis and a demo can be found at http://www.molgenis.org/connect.

## Supplementary Material

Supplementary Data
