# Supplementary material for: MOLGENIS/connect: a system for semi-automatic integration of heterogeneous phenotype data with applications in biobanks
Source: Bioinformatics. 2016 Mar 21;32(14):2176–83. doi: 10.1093/bioinformatics/btw155 (PMC4937195; doi:10.1093/bioinformatics/btw155)
Supplement: Supplementary Data [file supp_32_14_2176__index.html]

MOLGENIS/connect: a system for semi-automatic integration of heterogeneous phenotype data with applications in biobanks — MOLGENIS/connect: a system for semi-automatic integration of heterogeneous phenotype data with applications in biobanks — Supplementary Data 

# MOLGENIS/connect: a system for semi-automatic integration of heterogeneous phenotype data with applications in biobanks

## Supplementary Data

files

- Supplementary Data - doc file
