# Supplementary material for: MOLGENIS/connect: a system for semi-automatic integration of heterogeneous phenotype data with applications in biobanks
Source: Bioinformatics. 2016 Mar 21;32(14):2176–83. doi: 10.1093/bioinformatics/btw155 (PMC4937195; doi:10.1093/bioinformatics/btw155)
Supplement: Supplementary Data [file supp_btw155_bio_format_MolgenisConnect_supplementary.doc]

Databases and ontologies

# MOLGENIS/connect: a system for semi-automatic integration of heterogeneous phenotype data with applications in biobanks

Chao Pang^1,2^, David van Enckevort^1^, Mark de Haan^1^, Fleur Kelpin^1^, Jonathan Jetten^1^, Dennis Hendriksen^1^, Tommy de Boer^1^, Bart Charbon^1^, Erwin Winder^1^, Joeri K. van der Velde^1^, Dany Doiron^3^, Isabel Fortier^3^, Hans Hillege^2,*^, Morris A. Swertz^1,2,*^

^1^University of Groningen, University Medical Center Groningen, Genomics Coordination Center, Department of Genetics, Groningen, the Netherlands. ^2^University of Groningen, University Medical Center Groningen, Department of Epidemiology, Groningen, the Netherlands. ^3^Research Institute of the McGill University Health Centre and Department of Medicine, McGill University, Montreal, Canada.

# Supplementary Information

## Supplementary Table S1

**Matching amount categories**. Example of complex matches between target and source categories and the corresponding quantified amount that describes the frequency of potato consumption for the target attribute and the source attribute. First, the categories are converted to quantifiable amounts based on the key information (time unit and frequency) extracted from the description using regular expressions. Then, the source categories are matched to the target categories by determining the closest target amounts for source amounts.

| **Target**  **Current Consumption Frequency of Cooked Vegetables** | | **Source**  **Cooked vegetables** | |
| --- | --- | --- | --- |
| **Categories** | **Amounts** | **Categories** | **Amounts** |
| Never + less than once a week | Unit: week  Frequency: 0 - 1 | Less often than once a month or not at all | Unit: month  Frequency: 0 - 1 |
|  |  | 1-3 times a month | Unit: month  Frequency: 1 - 3 |
| About once a week | Unit: week  Frequency: 1 | Once a week | Unit: week  Frequency: 1 |
| Several times a week | Unit: week  Frequency: 2-7 | 2-4 times a week | Unit: week  Frequency: 2-4 |
|  |  | 5-6 times a week | Unit: week  Frequency: 5-6 |
| Almost daily + daily | Unit: day  Frequency: 1 | Once a day | Unit: day  Frequency: 1 |
|  |  | 2-3 times a day | Unit: day  Frequency: 2-3 |
|  |  | More than 4 times a day | Unit: day  Frequency: 4 |

## Supplementary Table S2

**Matching complex categories. Pre-defined rules for matching categories and example applications.**

| **Rule** | **Description** | **Example** | |
| --- | --- | --- | --- |
| Rule1 | Category label containing word ‘**No**’ can be matched to the category that contains ‘**Never**’ | No | Never had stroke |
| Rule2 | Category label containing word ‘**Yes**’ can be matched to the category that contains ‘**Ever**’ | Yes | Ever had stroke |
| Rule3 | Category label containing word ‘**Yes**’ can be matched to the category that contains ‘**Has**’ | Yes | Has had stroke |
| Rule4 | Category label containing word ‘**Unknown**’ can be matched to the category that contains ‘**Missing**’ | Unknown | Missing |
| Rule5 | Category label containing word ‘**Not know** can be matched to the category that contains ‘**Missing**’ | I do not know | Missing |

## Supplementary Figure S3

**The overview of the algorithm editor.**


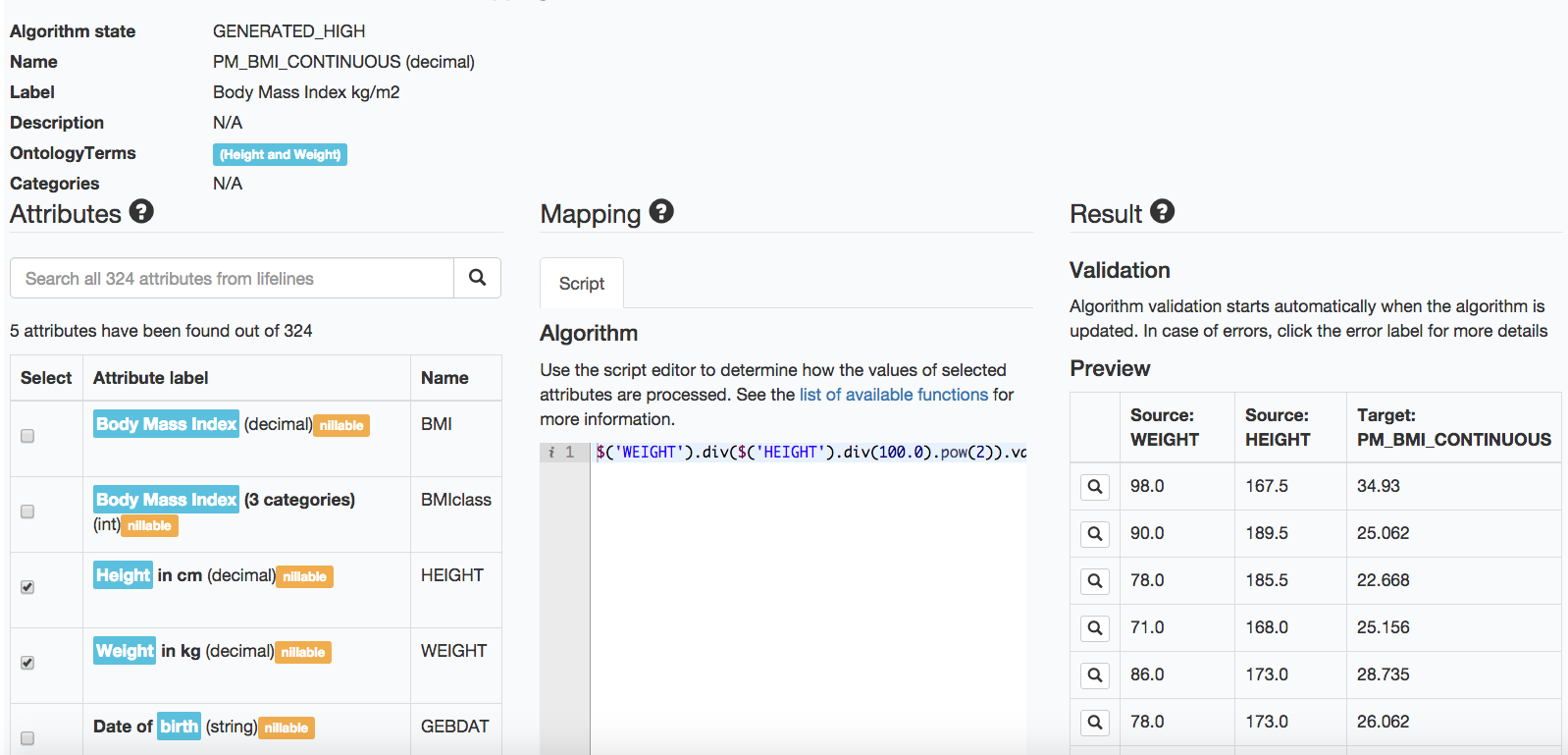


## Supplementary Figure S4

**Source attribute selector view** (A) The target attribute was automatically annotated with the ontology term ‘Hypertension’. All the synonyms and subclasses of ‘Hypertension’ were used for query expansion. Based on these, Lucene retrieved 13 relevant attributes from the LifeLines database source. The words Lucene used to match are highlighted. (B) The semantic search box allows the user to optionally search all source attributes. When a user types in a term, it will also be automatically annotated with ontology terms to enable query expansion as described above. The user-defined query terms have the highest priority and only these are used in semantic search. The attribute label, description and existing ontology term annotations will not be used for query expansion if there are user-defined queries.


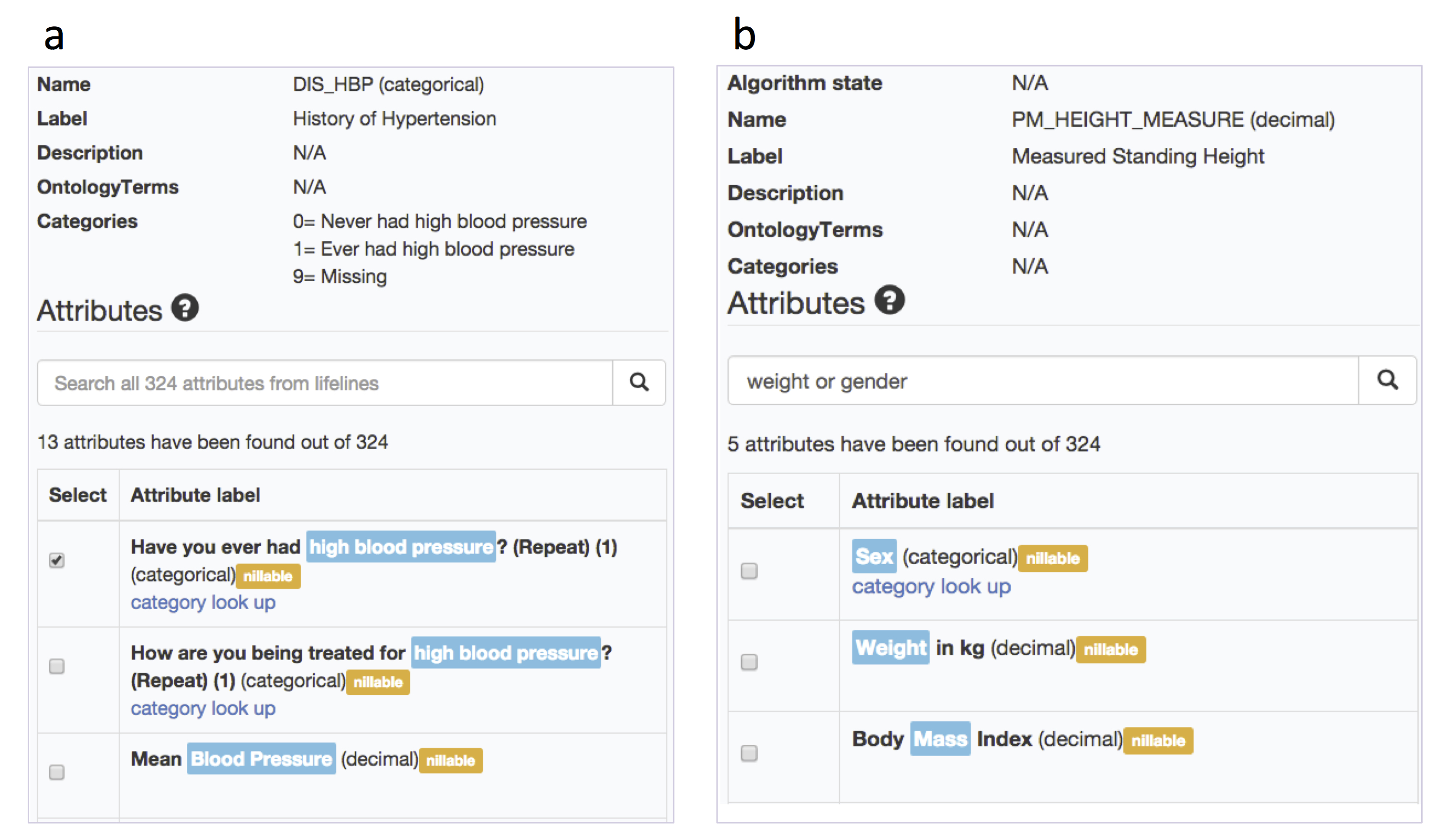


## Supplementary Figure S5

**Transformation algorithm editor** (A) The auto-generated algorithm for the target attribute ‘Measured Standing Height’ from source attribute ‘Height at physical examination (m)’. The mention of the unit **m** in the source attribute label is automatically detected and a unit (‘cm’) convertor added to algorithm. A preview of the algorithm conversion results is provided for the user to check. (B) Since the target attribute and the source attribute are both categorical, a category-matching editor is provided for the user to easily match categories using a user interface.


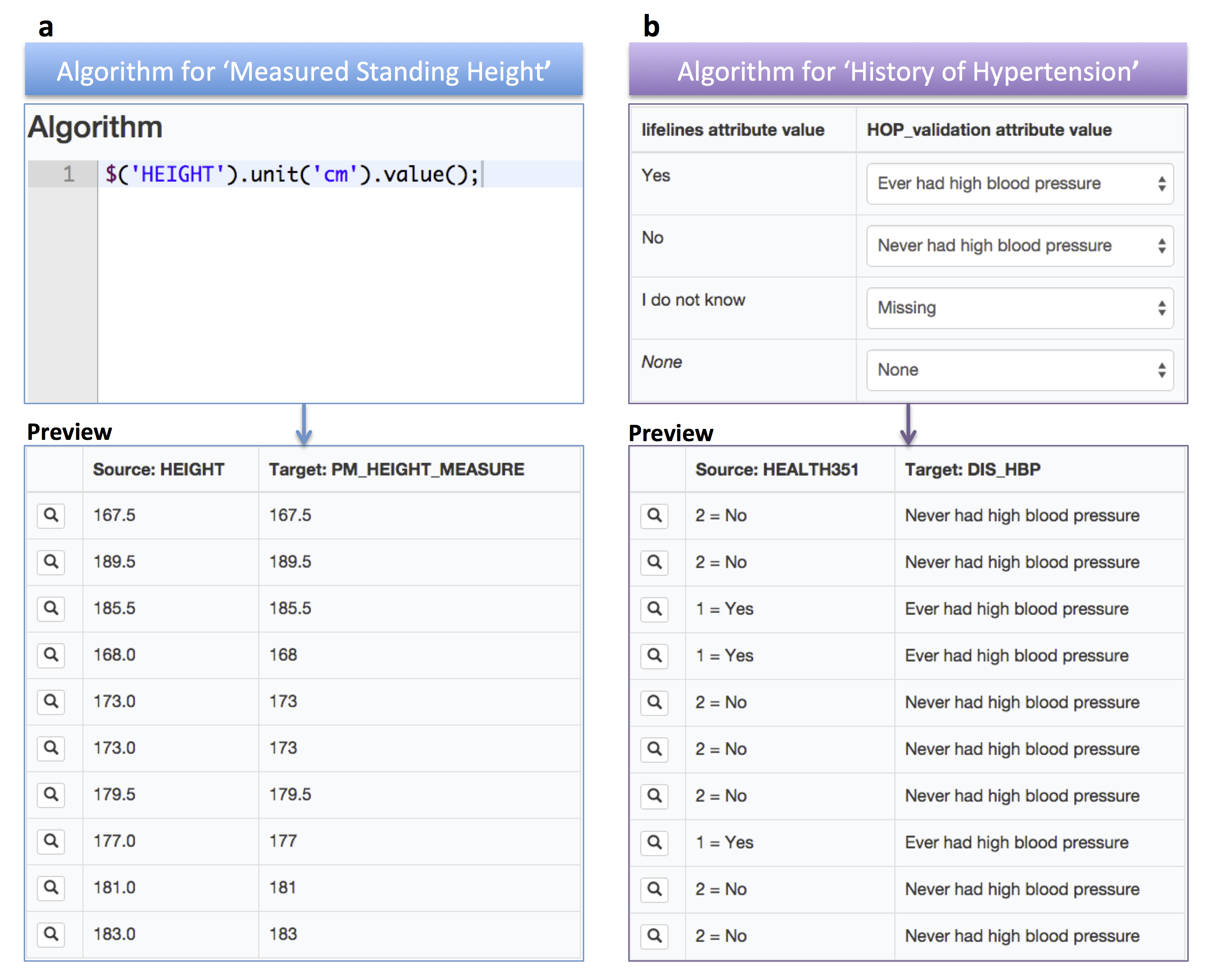


## Supplementary Table S6

**The summary of the evaluations of the semantic search and the algorithm generator**. For the algorithm generator, ‘Good’ means that the generated algorithms are either the same as or equivalent to the manually created algorithms; ‘Partially-good’ means that the generated algorithms are very similar to the manually created algorithms therefore can be easily fixed; ‘Bad’ that means the generated algorithms are very far from the manually created algorithms. For the semantic search, ‘Good’ that the attributes in the manually created algorithms are found within top 20 of the suggested data elements; ‘Bad’ that the attributes in the manually created algorithms are not found with top 20 of the suggested data elements.

|  | | **Prevend** | | **LifeLines** | | **Mitchelstown** | |
| --- | --- | --- | --- | --- | --- | --- | --- |
| **Topic** | **label** | **Algorithm generator** | **Semantic search** | **Algorithm generator** | **Semantic search** | **Algorithm generator** | **Semantic search** |
| Diet | Currently Follows a Cholesterol Lowering Diet | N/A | N/A | Partially-good | Perfect | Good | Perfect |
| Diet | Currently Follows a Diabetic Diet | N/A | N/A | Partially-good | Perfect | Good | Perfect |
| Diet | Currently Follows a Low Salt Diet | N/A | N/A | Partially-good | Perfect | N/A | N/A |
| Diet | Currently Follows a Non-Gluten Diet | N/A | N/A | N/A | N/A | Good | Perfect |
| Diet | Currently Follows a Vegetarian Diet | N/A | N/A | Bad | Good | Good | Good |
| Diet | Currently Follows a Weight Loss Diet | N/A | N/A | Partially-good | Good | Good | Perfect |
| Diet | Type of Vegetarian Diet | N/A | N/A | N/A | N/A | N/A | N/A |
| Disease | History of Diabetes | Good | Perfect | Good | Good | Good | Good |
| Disease | History of Hypertension | Good | Perfect | Good | Perfect | Good | Perfect |
| Disease | History of Myocardial Infarction | Good | Perfect | Good | Perfect | Good | Perfect |
| Disease | History of Stroke | Good | Perfect | Good | Perfect | Good | Perfect |
| Disease | Type of Diabetes | Partially-good | Good | Partially-good | Good | N/A | N/A |
| Drink | Current Quantity of Beer Consumed | N/A | N/A | N/A | N/A | Bad | Perfect |
| Drink | Current Quantity of Spirits/Liquor Consumed | N/A | N/A | Bad | Good | Bad | Bad |
| Drink | Current Quantity of Total Alcohol Consumed in Beer, Wine and Spirits per week | N/A | N/A | N/A | N/A | N/A | N/A |
| Drink | Current Quantity of Wine Consumed | N/A | N/A | Bad | Good | Bad | Perfect |
| Drink | Current Use of Alcohol | Partially-good | Perfect | Partially-good | Good | Partially-good | Perfect |
| Drink | Level of current alcohol consumption | N/A | N/A | N/A | N/A | N/A | N/A |
| Education | Highest Level of Education | Bad | Good | Bad | Good | Partially-good | Perfect |
| Education | Highest Level of Education | N/A | N/A | N/A | N/A | N/A | N/A |
| Education | Number of Years of Education | Bad | Perfect | N/A | N/A | Partially-good | Perfect |
| Education | Some Elements of Post-Secondary Non-Tertiary Education Completed | Partially-good | Perfect | Partially-good | Good | Partially-good | Perfect |
| Education | Some Elements of Tertiary Education Completed | Partially-good | Perfect | Partially-good | Good | Partially-good | Perfect |
| Education | Some Primary Education Completed | Partially-good | Perfect | Partially-good | Good | Partially-good | Perfect |
| Education | Some Secondary Education Completed | Partially-good | Perfect | Partially-good | Good | Partially-good | Perfect |
| Food | Current Consumption Frequency of Bakery Products | N/A | N/A | N/A | N/A | Good | Bad |
| Food | Current Consumption Frequency of Bread and Rolls | N/A | N/A | Good | Bad | Good | Bad |
| Food | Current Consumption Frequency of Breakfast Cereals | N/A | N/A | Good | Good | Good | Bad |
| Food | Current Consumption Frequency of Cheese | N/A | N/A | Good | Good | Good | Good |
| Food | Current Consumption Frequency of Chocolate | N/A | N/A | Good | Bad | Good | Bad |
| Food | Current Consumption Frequency of Chocolates/Sweets | N/A | N/A | Good | Bad | Good | Bad |
| Food | Current Consumption Frequency of Cooked Vegetables | N/A | N/A | Good | Good | N/A | N/A |
| Food | Current Consumption Frequency of Eggs | N/A | N/A | Good | Perfect | Good | Perfect |
| Food | Current Consumption Frequency of Fish | N/A | N/A | Good | Perfect | Good | Good |
| Food | Current Consumption Frequency of Fruits | N/A | N/A | Good | Perfect | Good | Good |
| Food | Current Consumption Frequency of Meat and Meat Products | N/A | N/A | Good | Bad | Good | Bad |
| Food | Current Consumption Frequency of Milk | N/A | N/A | Good | Good | Bad | Bad |
| Food | Current Consumption Frequency of Nuts | N/A | N/A | Good | Good | Good | Perfect |
| Food | Current Consumption Frequency of Potatoes | N/A | N/A | Good | Good | Good | Good |
| Food | Current Consumption Frequency of Poultry and Poultry Products | N/A | N/A | N/A | N/A | Good | Bad |
| Food | Current Consumption Frequency of Raw Vegetables | N/A | N/A | Good | Good | N/A | N/A |
| Food | Current Consumption Frequency of Rice and Pasta | N/A | N/A | Good | Good | Good | Bad |
| Food | Current Consumption Frequency of Salted Snacks | N/A | N/A | Good | Bad | Good | Bad |
| Food | Current Consumption Frequency of Soft Drinks | N/A | N/A | Good | Good | Good | Bad |
| Food | Current Consumption Frequency of Sugar Products Excluding Chocolate | N/A | N/A | Good | Bad | Bad | Bad |
| Food | Current Consumption of Milk Products | N/A | N/A | Good | Bad | Bad | Bad |
| Food | Current Consumption Quantity of Coffee | N/A | N/A | Partially-good | Bad | Good | Good |
| Food | Current Consumption Quantity of Tea | N/A | N/A | Partially-good | Bad | Good | Perfect |
| General | Age in Years | Bad | Bad | Bad | Bad | Good | Perfect |
| General | Birth Year | Partially-good | Perfect | Partially-good | Perfect | Partially-good | Good |
| General | Country of Birth | N/A | N/A | Partially-good | Good | N/A | N/A |
| General | Current Country of Residence | Good | Perfect | N/A | N/A | N/A | N/A |
| General | Current Region of Residence | N/A | N/A | N/A | N/A | N/A | N/A |
| General | Gender | Good | Perfect | Good | Perfect | Good | Perfect |
| General | Living with Partner | Bad | Bad | N/A | N/A | Partially-good | Bad |
| General | Marital Status | N/A | N/A | N/A | N/A | Partially-good | Perfect |
| General | Net Household Income | N/A | N/A | N/A | N/A | N/A | N/A |
| General | Number of Live Births Mothered | N/A | N/A | N/A | N/A | Bad | Bad |
| General | Number of People in the Household | N/A | N/A | N/A | N/A | Partially-good | Perfect |
| General | Year of Interview | Partially-good | Bad | Partially-good | Bad | N/A | N/A |
| Job | Current Job Title (ISCO 88) | N/A | N/A | N/A | N/A | Partially-good | Bad |
| Job | Employment Status | Partially-good | Perfect | Partially-good | Bad | Partially-good | Bad |
| Job | Number of Working Hours | N/A | N/A | Partially-good | Perfect | N/A | N/A |
| Job | Retirement Status | Partially-good | Bad | Partially-good | Bad | N/A | N/A |
| Job | Student Status | N/A | N/A | Partially-good | Bad | N/A | N/A |
| Measurement | Body Mass Index kg/m2 | Good | Perfect | Good | Perfect | Good | Perfect |
| Measurement | Creatinin | Bad | Bad | Good | Perfect | Good | Perfect |
| Measurement | Fasting Glucose | Bad | Bad | Good | Perfect | Partially-good | Perfect |
| Measurement | HDL Cholesterol | Good | Perfect | Good | Perfect | Good | Bad |
| Measurement | Hip Circumference | Good | Perfect | Good | Perfect | Bad | Good |
| Measurement | Inflammation Marker (hsCRP) | Good | Perfect | Good | Perfect | Partially-good | Perfect |
| Measurement | LDL Cholesterol (Friedewald Equation) | N/A | N/A | N/A | N/A | N/A | N/A |
| Measurement | Measured Diastolic Blood Pressure | Good | Perfect | Partially-good | Perfect | Bad | Good |
| Measurement | Measured Standing Height meter | N/A | N/A | Good | Perfect | Partially-good | Perfect |
| Measurement | Measured Systolic Blood Pressure | Good | Perfect | Partially-good | Perfect | Bad | Good |
| Measurement | Measured Weight kilogram | Good | Perfect | Good | Perfect | Good | Perfect |
| Measurement | Microalbuminuria | Bad | Bad | Bad | Bad | Partially-good | Bad |
| Measurement | Non-Fasting Glucose | Bad | Bad | N/A | N/A | N/A | N/A |
| Measurement | Total Serum Cholesterol | Good | Perfect | Good | Perfect | Partially-good | Perfect |
| Measurement | Triglycerides | Good | Perfect | Good | Perfect | Good | Perfect |
| Measurement | Waist Circumference | Good | Perfect | Good | Perfect | Bad | Good |
| Medication | Current Use of Antihypertensive Medication | Partially-good | Good | Bad | Good | Partially-good | Perfect |
| Medication | Current Use of Blood Glucose Lowering Medication | Partially-good | Perfect | Bad | Bad | Bad | Bad |
| Medication | Current Use of Lipid Lowering Medication | Partially-good | Perfect | Bad | Bad | Bad | Bad |
| Medication | Current Use of Lipid Lowering Medications Fibrates and/or Nicotinic Acid Derivatives | N/A | N/A | Bad | Good | Bad | Good |
| Smoking | Current Cigar Smoker | N/A | N/A | Bad | Bad | Bad | Good |
| Smoking | Current Cigarette Smoker | N/A | N/A | Bad | Bad | Bad | Good |
| Smoking | Current Pipe Smoker | N/A | N/A | Bad | Bad | Bad | Good |
| Smoking | Current Quantity of Cigarettes Smoked | Bad | Good | Bad | Bad | N/A | N/A |
| Smoking | Current Tobacco Smoker | Partially-good | Perfect | N/A | N/A | Partially-good | Good |
| Smoking | Ever Smoked Cigarettes | N/A | N/A | N/A | N/A | Bad | Good |
| Smoking | Ever Smoked Tobacco | Good | Good | N/A | N/A | Partially-good | Good |
| Smoking | Smoking status | N/A | N/A | N/A | N/A | Good | Perfect |

## Supplementary material external files

One Microsoft Excel file Evaluation_results_complete.xlsx, javascript_magma.xls can be found at <https://molgenis26.target.rug.nl/downloads/molgenis-connect/supplementary_material/>
